# Emergency Department Use by Centenarians: The 2008 Nationwide Emergency Department Sample

**DOI:** 10.5888/pcd10.120006

**Published:** 2013-11-27

**Authors:** Matthew R. Carey, Embry M. Howell, Megan Colleen McHugh

**Affiliations:** Author Affiliations: Embry M. Howell, Health Policy Center, the Urban Institute, Washington, DC; Megan Colleen McHugh, Center for Healthcare Studies and Department of Emergency Medicine, Northwestern University, Feinberg School of Medicine, Chicago, Illinois.

## Abstract

**Introduction:**

Older adults have higher rates of emergency department use than do younger adults, and the number of centenarians is expected to increase. The objective of this study was to examine centenarians’ use of the emergency department in the United States, including diagnoses, charges, and disposition.

**Methods:**

The 2008 Nationwide Emergency Department Sample, Healthcare Cost and Utilization Project, Agency for Healthcare Research and Quality provided encounter-level data on emergency department visits and weights for producing nationwide estimates. From this data set, we collected patient characteristics including age, sex, primary diagnosis, and disposition. We used χ^2^ tests and *t* tests to test for significant differences among people aged 80 to 89, 90 to 99, and 100 years or older.

**Results:**

Centenarians had a lower rate of emergency department use than those aged 90 to 99 (736 per 1,000 vs 950 per 1,000; *P* < .05). We found no significant difference in use between centenarians and those aged 80 to 89. The most common diagnoses for centenarians were superficial injuries (5.8% of visits), pneumonia (5.1%), and urinary tract infections (5.1%). Centenarians were more likely to visit the emergency department for fall-related injuries (21.5%) than those aged 80 to 89 (14.1%; *P* < .05) and 90 to 99 (18.7%; *P* < .05). Centenarians were more likely to die in the emergency department (2.0%) than were those aged 80 to 89 (0.6%; *P* < .05) and 90 to 99 (0.7%; *P* < .05).

**Conclusion:**

Centenarians in emergency departments in the United States have different diagnoses, conditions, and outcomes than other older Americans.

## Introduction

The number of centenarians in the United States is projected to increase more than 700% during the coming decades, from 79,000 in 2008 to more than 600,000 in 2050 (1). Although in absolute terms these increases are small, they indicate that this minority group may one day strongly influence health policy. This growth has led to research on the health outcomes of these eldest members of society (2–10). Studies have noted the unique health care needs and use patterns of centenarians compared with other older populations. Many centenarians, for instance, have been able to delay or avoid chronic conditions commonly associated with old age such as heart disease and stroke (8). Centenarians also have lower instances of cancer (9,10). Despite this body of literature on centenarians and many studies on emergency department use by older adults in general, there is little research on emergency department use by centenarians (11–13).

Understanding the patterns of use and etiology of emergency department visits among centenarians is important for several reasons. First, emergency physician and nurse training can use this information to tailor services to the needs of this population, which will represent a greater share of visits in the future. Second, hospital administrators can use this information for capacity planning, which is crucial to the problem of crowding in emergency departments nationwide (14). This problem may be exacerbated by the growing number of centenarians who may be more likely to be admitted to the hospital. Third, this information may be used to develop targeted services to prevent emergency department visits by centenarians. Understanding why centenarians visit the emergency department will also allow health care administrators to determine the appropriateness of such visits and design programs that could address the primary causes of emergency department use among this group, thereby reducing the number of visits and slowing the rise of health care costs. The objective of this study was to describe centenarians’ use of emergency department services, including rates of use, primary diagnoses, charges, and disposition.

## Methods

We used the 2008 Nationwide Emergency Department Sample (NEDS) to collect data on emergency department use by the oldest old (15). NEDS is a nationwide data set created as part of the Healthcare Cost and Utilization Project (HCUP), a federal–state–industry partnership sponsored by the Agency for Healthcare Research and Quality (AHRQ) (16). As the largest emergency department data set available, it is useful for studying emergency department use by centenarians, given that they comprise such a small percentage of the population. The data set is a 20%-stratified sample of 980 emergency departments in 24 states in the United States (16). NEDS provides information on disposition, diagnoses, procedures, charges, patient characteristics (eg, age, sex, geographic residence [Northeast, Midwest, South, and West]), and hospital emergency department characteristics (eg, trauma center, urban/rural location, region, teaching status) for more than 25 million visits annually (16). From this sample, researchers can create nationwide estimates of emergency department visits by using discharge weights provided in NEDS (16).

For this study, we grouped emergency department visits according to age, sex, US Census region, urban or rural residence of the patient, disposition from the emergency department, and primary diagnosis. NEDS uses the 6-category urban/rural classification system designed by the National Center for Health Statistics (NCHS), which can be mapped onto the United States Department of Agriculture’s 2003 Urban Influence Codes (UICs) to classify patients’ residences as urban or rural (16). We defined a patient’s residence as urban if the person’s county of residence had an NCHS code of 1 to 4, corresponding to a UIC of 1 or 2 (16). These 2 UICs encompass all metropolitan counties in the United States, as defined by the Office of Management and Budget’s metro/non-metro county categorical system (17). We classified patients living in counties with any other UICs as rural patients. We calculated average emergency department charges for each age cohort based on the charge data provided in NEDS for each visit. NEDS lacks information on emergency department charges for 18% of emergency department visits (16). Observations that lacked data on emergency department charges were excluded when we calculated average emergency department charges for each age cohort. Although NEDS provides ICD-9-CM (*International Classification of Diseases, Ninth Revision, Clinical Modification*) codes for up to 15 diagnoses per emergency department visit, we used only the first diagnosis listed for this analysis. According to AHRQ, the first-listed diagnosis for an emergency department record indicates the “code for the diagnosis, condition, problem or other reason for encounter/visit shown in the medical record to be chiefly responsible for the services provided” (18). We applied the Clinical Classifications Software (CCS) — a means of grouping ICD-9-CM codes developed as part of the HCUP — to the primary diagnosis ICD-9-CM codes found in the NEDS to group these diagnoses into meaningful categories (19). We identified the 15 most common primary admitting diagnoses in our sample. In addition to the diagnostic codes, NEDS provides separate external cause-of-injury codes for people who visit the emergency department because of injury. We used the CCS code E2603 to determine the number of patients visiting the emergency department because of falls.

To produce the population denominators needed for constructing use rates, we used the 2008 Population Projections created by the US Census Bureau (20). We were not able to use population estimates derived from national surveys such as the American Community Survey or Current Population Survey because their small sample sizes do not allow for accurate estimates of the number of centenarians. The 2008 Population Projections provided total population estimates and sex-specific estimates for each of the age cohorts used in the study: aged 80 to 89 years, aged 90 to 99 years, and aged 100 years or older (centenarians). Because these population projections do not provide detailed information on the US population by census region or urban/rural residence, we applied the proportions from the 2000 Census Summary File 4 data set to the 2008 population projections (21). Our assumption was that older adults have a low rate of migration and that any bias caused by this assumption would be limited (22). The NEDS urban/rural classification system for patients was also used to determine the urban and rural population totals for each age cohort.

We used SAS 9.2 (SAS Institute Inc, Cary, North Carolina) for all data analyses. Because of the complex sampling design and weighting used in NEDS, the NEDS documentation provides detailed information on variance calculations necessary for constructing confidence intervals and testing for significance. The supplementary hospital file allows standard errors to be calculated for subsets of NEDS (23). We used χ^2^ tests to test for significant differences in the distribution of dispositions from the emergency department and diagnoses by age cohort. We used *t* tests to test for significant differences in the rates of emergency department use by age cohort. For all tests, we used *P* < .05 as the significance level. Because we made multiple comparisons when analyzing the dispositions and diagnoses of the 3 cohorts, we applied the Bonferroni correction to the significance level, using *P* < .0083 as the adjusted significance level when comparing dispositions and *P* < .0033 as the adjusted significance level when comparing diagnoses, both of which are equivalent to *P* < .05 for a single test.

## Results

In 2008 nationwide, there were an estimated 7,061,763 emergency department visits among people aged 80 to 89; 1,786,792 visits among those aged 90 to 99; and 52,265 visits among those aged 100 or older. The overall use rate (Table 1) for centenarians was significantly lower than the rate for people aged 90 to 99 (736 per 1,000 vs 950 per 1,000). We found no significant difference between the use rates of centenarians and those aged 80 to 89 (736 per 1,000 vs 766 per 1,000). We found a significant difference between use rates of male and female centenarians (952 per 1,000 vs 687 per 1,000). This difference was much larger than the difference for the other 2 age cohorts: 766 per 1,000 for both sexes aged 80 to 89 and 987 per 1,000 for men and 936 per 1,000 for women aged 90 to 99. We found no significant difference between the use rates of centenarians and those aged 80 to 89 for any of the 4 geographic areas. Centenarians had fewer visits per 1,000 than those aged 90 to 99 in the Northeast (705 per 1,000 vs 922 per 1,000) and South (789 per 1,000 vs 997 per 1,000).

**Table 1:** Emergency Department Visit Rate per 1,000 for People Aged 80 to 89, 90 to 99, and 100 Years or Older, by Sex, Region, and Urban/Rural Designation,^a^ United States, 2008

Population Group	Visit Rate per 1,000 (95% Confidence Interval)		
	Age 80–89 (n = 7,061,763)^b^	Age 90–99 (n = 1,786,792)^b^	Age ≥100 (n = 52,265)^b^
Total	766 (726–806)	950^c^ (899–1,002)	736 (688–783)
**Sex**			
Male	766^c^ (724–808)	987 (924–1,050)	952 (837–1,067)
Female	766 (727–805)	936^c^ (887–985)	687 (645–729)
**Region**			
Northeast	705 (625–785)	922^c^ (816–1,028)	705 (612–798)
Midwest	735 (666–803)	882 (799–964)	677 (693–870)
South	824 (747–902)	997^c^ (897–1,098)	789 (693–884)
West	773 (686–860)	995 (869–1,121)	755 (641–870)
**Urban/rural designation**			
Urban	770 (722–818)	971^c^ (907–1,035)	743 (690–795)
Rural	739^c^ (693–784)	865^c^ (807–923)	619 (555–684)

a The 2008 Nationwide Emergency Department Sample (15) was used to estimate the number of visits. The United States Census 2000: Summary File 4 (21), 2008 Population Projections (20), and US Department of Agriculture Urban Influence Codes (16) were used to estimate the population figures needed to derive rates.

b Weighted number of emergency department visits for each age cohort.

c Significantly different from rate for those aged ≥100 (*P* < .05).

All age cohorts had more visits per 1,000 in urban areas than in rural areas, but these differences were significant only for centenarians (743 per 1,000 vs 619 per 1,000). Those aged 90 to 99 had significantly higher use rates than centenarians in both urban (971 per 1,000 vs 743 per 1,000) and rural (865 per 1,000 vs 619 per 1,000) areas. When we compared use rates between those aged 80 to 89 and those aged 100 or older, we found a significant difference only for rural areas: 739 per 1,000 for those aged 80 to 89 and 619 per 1,000 for those aged 100 or older.

Although centenarians had the highest emergency department charges per visit, the differences among the age cohorts were not significant. Centenarians had average emergency department charges of $2,227 per visit, compared with $2,123 for those aged 80 to 89 and $2,032 for those aged 90 to 99.

Significantly more centenarians were transferred to a skilled nursing facility (6.9%) than were those aged 90 to 99 (5.4%) or 80 to 89 (3.1%) (Table 2). In addition, significantly more centenarians died in the emergency department (2.0%) than did those aged 90 to 99 (0.7%) or 80 to 89 (0.6%).

**Table 2:** Disposition From the Emergency Department for People Aged 80 to 89, 90 to 99, and 100 Years or Older,^a^ United States, 2008

Disposition from the Emergency Department	Percentage of Total Visits for Each Age Cohort		
	Age 80–89 (n = 7,061,763)^b^	Age 90–99 (n = 1,786,792)^b^	Age ≥100 (n = 52,265)^b^
Admitted or transferred to another hospital	49.0	53.3^c^	50.7
Transferred to a skilled nursing facility	3.1^c^	5.4^c^	6.9
Transferred to home health care	0.3	0.4	0.4
Discharged	46.2^c^	39.7	38.5
Died	0.6^c^	0.7^c^	2.0
Other (against medical advice or unknown)	0.9^c^	0.6^c^	1.4

a The 2008 Nationwide Emergency Department Sample (15) was used to estimate the number of emergency department visits by age cohort and disposition.

b Weighted number of emergency department visits for each age cohort.

c Significantly different from the percentage for those aged ≥100 at a .05 level; because multiple comparisons were made, we applied the Bonferroni correction, using *P* < .0083 as the adjusted significance level.

We found significant differences by age cohort in the 15 most common primary admitting diagnoses (Table 3). The most common diagnosis for centenarians was superficial injury or contusion (5.8%), whereas the most common diagnosis for those aged 80 to 89 and 90 to 99 year was congestive heart failure (nonhypertensive). Other common diagnoses for centenarians were pneumonia (5.1%) and urinary tract infections (5.1%). Centenarians had a significantly lower percentage of visits for cardiac dysrhythmias (1.8%), nonspecific chest pain (1.8%), syncope (1.7%), and chronic obstructive pulmonary disease and bronchiectasis (1.1%) than those aged 80 to 89 and 90 to 99. According to the external cause-of-injury code for falls, centenarians had a significantly greater percentage of visits resulting from falls (21.5%) than did those aged 90 to 99 (18.7%) or 80 to 89 (14.1%).

**Table 3:** Percentage Distribution of Emergency Department Visits, by Age and Primary Admitting Diagnosis,^a^ United States, 2008

Primary Admitting Diagnosis (CCS Identifier)^b^	Percentage of Total Visits for Each Age Cohort		
	Age 80–89 (n = 7,061,763)^c^	Age 90–99 (n = 1,786,792)^c^	Age ≥100 (n = 52,265)^c^
Superficial injury, contusion (239)	3.9^d^	4.7^d^	5.8
Pneumonia (except that caused by tuberculosis or sexually transmitted disease) (122)	3.5^d^	4.4^d^	5.1
Urinary tract infections (159)	3.9^d^	4.8	5.1
Congestive heart failure, nonhypertensive (108)	4.1^d^	5.4	4.9
Open wounds of head, neck, and trunk (235)	1.7^d^	2.2^d^	3.8
Hip fracture (226)	1.9^d^	3.0	3.1
Septicemia (2)	2.4^d^	3.0	3.0
Fluid and electrolyte disorders (55)	2.0	2.4	2.4
Acute cerebrovascular disease (109)	2.1	2.3^d^	1.8
Cardiac dysrhythmias (106)	3.0^d^	2.6^d^	1.8
Nonspecific chest pain (102)	3.2^d^	2.4^d^	1.8
Syncope (245)	2.3^d^	2.2^d^	1.7
Abdominal pain (251)	1.8^d^	1.4	1.2
COPD and bronchiectasis (127)	2.7^d^	1.8^d^	1.1
Spondylosis, intervertebral disc disorders, back problems (205)	1.8^d^	1.5^d^	1.1
All other primary diagnoses	59.7	56.2	56.3
Total diagnoses	100.0	100.0	100.0
Falls (E2603)^e^	14.1^d^	18.7^d^	21.5

Abbreviations: CCS, Clinical Classifications Software; COPD, chronic obstructive pulmonary disease.

a The 2008 Nationwide Emergency Department Sample (NEDS) (15) was used to estimate the number of visits by age cohort and primary diagnosis.

b The Nationwide Emergency Department Sample uses ICD-9-CM codes to label the admitting diagnoses for all patients (16). We applied the Clinical Classifications Software, a means of grouping ICD-9-CM codes, to create meaningful categories (19).

c Weighted number of emergency department visits for each age cohort.

d Significantly different from the percentage for those aged ≥100 at a level of .05; because multiple comparisons were made, we applied the Bonferroni correction, using *P* < .0033 as the adjusted significance level.

e NEDS provides separate external cause-of-injury codes for people who visit the emergency department because of injury. We used CCS code E2603 to determine the number of patients visiting the emergency department because of falls.

## Discussion

Although centenarians had lower rates of emergency department use than other older persons, a significantly greater proportion of centenarians died in the emergency department. The elderly visit the emergency department for different reasons than younger people (12). Our study indicated differences in patterns of emergency department use in the elderly cohort — specifically, differences between centenarians and those aged 80 to 89 and 90 to 99. The distribution of primary diagnoses indicates that centenarians visit the emergency department with needs that are different from those of the younger 2 age cohorts.

Other research on the health status of centenarians and the oldest old may give clues as to the causes for these differences in emergency department use. Some studies show that centenarians seem to delay or avoid typical age-related diseases and disorders, including cancer (8–10). The ability to reach extreme ages while avoiding common chronic conditions may explain why centenarians are less likely to visit the emergency department for cardiac dysrhythmias, chronic obstructive pulmonary disease, chest pain, and acute cerebrovascular disorders. Other studies, however, found high rates of certain chronic conditions, such as hypertension and heart disease, so no definitive conclusions can be made (24,25).

Although many centenarians may have avoided chronic conditions, they are not immune from the frailty associated with old age, which may increase the risk of infections such as pneumonia and influenza (26). This increased susceptibility to infection is consistent with the findings of our study, which shows that centenarians have proportionally more emergency department visits for pneumonia than those aged 80 to 89 or 90 to 99.

Our study indicates that centenarians are more likely than those aged 80 to 89 or 90 to 99 to visit the emergency department for falls. Higher proportions of visits for “superficial injury, contusion” and “open wounds of head, neck, and trunk” among people aged 100 or older than among the younger 2 age cohorts also support this finding. One study found that centenarians have low scores on measures of daily social and productive functioning (27). Another study found a correlation between a higher prevalence of fractures and a low level of functional ability among centenarians, although the authors cautioned that this finding may be only an artifact of the increased susceptibility of elderly people to sustaining fractures (25). Taken together, our data on emergency department visits for centenarians conforms to the findings of other research on the health characteristics of centenarians.

Our study has several limitations. First, because the 2008 NEDS data set provides information only on about half of all emergency department visits and then weights these to create national estimates, the use rates calculated in our study may have over- or underestimated the true rates. This limitation was particularly worrisome when we examined the number of emergency department visits among centenarians. Because of their small fraction of the US population, centenarians likely are not distributed equally among areas included in NEDS and areas outside the NEDS survey area; this unequal distribution may have led to inaccurate national estimates when we applied the discharge weights. The second major limitation of this study is related to the construction of population denominators for the rates of emergency department use. The total population for each age cohort was based on projections produced by the US Census Bureau rather than estimates from a national survey conducted in 2008. This method could have led to inaccurate estimates of total populations for each age cohort, especially centenarians, because of the rapid growth of this age cohort. Using the regional population distributions from the 2000 Census to calculate the regional population for each age cohort added another potential source of error.

The NEDS database has several other limitations that constrained the number of analyses that could be conducted. First, NEDS provides encounter-level, rather than patient-level, data (16). Therefore, we could not use the NEDS to follow individual patients and determine how many visits were by the same person. Second, although NEDS provides the patient’s destination after discharge, it provides no information on where the patient was before arriving at the emergency department (16). As a result, one relevant analysis — how many patients arrived from a skilled nursing facility — could not be performed. These limitations could be addressed by using emergency department data from hospitals, at the expense of the large sample size of centenarians found in NEDS, and may be an area for future research. Our research also indicates only whether centenarians are more likely to go to the emergency department for a particular condition, not whether they are more likely to contract that condition.

As the elderly population grows and hospital emergency departments become more crowded, understanding patterns of emergency department use among the oldest Americans is important. This research fills a void in the literature by analyzing the frequency, diagnoses, and charges for emergency department visits among the oldest old. Centenarians attend the emergency department for different conditions and have worse outcomes (ie, higher death rates) vis-à-vis slightly younger people. Although additional research must be done on the appropriateness of these visits as well as the relationship between the prevalence of a condition in the centenarian population and the number who visit the emergency department for that condition, our study indicates that it may be necessary to view this population as distinct from slightly younger people (ie, those aged 80 to 99) when characterizing emergency department use. As the centenarian population grows, health systems engineers and health care providers responsible for organizing and providing care in the emergency department will be able to use this and future research to ensure that the necessary equipment and properly trained staff are available to treat this population. Understanding the reasons for and outcomes of centenarians’ visits to the emergency department will be necessary to inform discussions on efficient yet ethical allocations of these resources in emergency departments nationwide.
